# In Vitro Cytotoxicity and In Vivo Antitumor Activity of Lipid Nanocapsules Loaded with Novel Pyridine Derivatives

**DOI:** 10.3390/pharmaceutics15061755

**Published:** 2023-06-16

**Authors:** Amr Selim Abu Lila, Mohammed Amran, Mohamed A. Tantawy, Ehssan H. Moglad, Shadeed Gad, Hadil Faris Alotaibi, Ahmad J. Obaidullah, El-Sayed Khafagy

**Affiliations:** 1 Department of Pharmaceutics, College of Pharmacy, University of Hail, Hail 81442, Saudi Arabia; a.abulila@uoh.edu.sa; 2 Department of Pharmaceutics and Industrial Pharmacy, Faculty of Pharmacy, Zagazig University, Zagazig 44519, Egypt; 3 Department of Pharmacy, Faculty of Health Sciences, Thamar University, Thamar 87246, Yemen; mohamran2013@gmail.com; 4 Department of Pharmacy, Al-Manara College for Medical Sciences, Maysan 62001, Iraq; 5 Hormones Department, Medical Research and Clinical Studies Institute, National Research Centre, Dokki, Giza 12622, Egypt; mohamed_tantawy@daad-alumni.de; 6 Stem Cells Lab, Center of Excellence for Advanced Sciences, National Research Centre, Dokki, Giza 12622, Egypt; 7 Department of Pharmaceutics, College of Pharmacy, Prince Sattam Bin Abdulaziz University, Al-Kharj 11942, Saudi Arabia; e.moglad@psau.edu.sa; 8 Department of Microbiology and Parasitology, Medicinal and Aromatic Plants Research Institute, National Center for Research, Khartoum 2404, Sudan; 9 Department of Pharmaceutics and Industrial Pharmacy, Faculty of Pharmacy, Suez Canal University, Ismailia 41522, Egypt; shaded_abdelrahman@pharm.suez.edu.eg; 10 Department of Pharmaceutical Sciences, College of Pharmacy, Princess Nourah Bint Abdul Rahman University, Riyadh 11671, Saudi Arabia; hfalotaibi@pnu.edu.sa; 11 Department of Pharmaceutical Chemistry, College of Pharmacy, King Saud University, Riyadh 11451, Saudi Arabia; aobaidullah@ksu.edu.sa

**Keywords:** antitumor activity, Ehrlich ascites carcinoma, MTT assay, nanocapsule, pyridine derivatives

## Abstract

This study demonstrates high drug-loading of novel pyridine derivatives (S1–S4) in lipid- and polymer-based core–shell nanocapsules (LPNCs) for boosting the anticancer efficiency and alleviating toxicity of these novel pyridine derivatives. The nanocapsules were fabricated using a nanoprecipitation technique and characterized for particle size, surface morphology, and entrapment efficiency. The prepared nanocapsules exhibited a particle size ranging from 185.0 ± 17.4 to 223.0 ± 15.3 nm and a drug entrapment of >90%. The microscopic evaluation demonstrated spherical-shaped nanocapsules with distinct core–shell structures. The in vitro release study depicted a biphasic and sustained release pattern of test compounds from the nanocapsules. In addition, it was obvious from the cytotoxicity studies that the nanocapsules showed superior cytotoxicity against both MCF-7 and A549 cancer cell lines, as manifested by a significant decrease in the IC_50_ value compared to free test compounds. The in vivo antitumor efficacy of the optimized nanocapsule formulation (S4-loaded LPNCs) was investigated in an Ehrlich ascites carcinoma (EAC) solid tumor-bearing mice model. Interestingly, the entrapment of the test compound (S4) within LPNCs remarkably triggered superior tumor growth inhibition when compared with either free S4 or the standard anticancer drug 5-fluorouracil. Such enhanced in vivo antitumor activity was accompanied by a remarkable increase in animal life span. Furthermore, the S4-loaded LPNC formulation was tolerated well by treated animals, as evidenced by the absence of any signs of acute toxicity or alterations in biochemical markers of liver and kidney functions. Collectively, our findings clearly underscore the therapeutic potential of S4-loaded LPNCs over free S4 in conquering EAC solid tumors, presumably via granting efficient delivery of adequate concentrations of the entrapped drug to the target site.

## 1. Introduction

Cancer is a worldwide health problem characterized by the abnormal proliferation of malignant cells with the ability to infiltrate or spread to other regions of the body [1]. It is regarded as the second leading cause of mortality globally, after cardiovascular disease, and it overtakes cardiovascular disease as the leading cause of mortality in high-income countries [2]. More than 10 million new cases of cancer are diagnosed annually, and the World Health Organization forecasts that cancer-related deaths will grow to over 13.1 million by 2030, with the greatest rise in low- and middle-income countries [3]. Despite the fact that cancer-related mortality has declined in recent years due to a better understanding of tumor biology, which has led to remarkable progress in cancer detection, prevention, and treatment, the development of new anticancer agents that show higher therapeutic efficacies along with minimized adverse effects remains the ultimate goal in cancer research.

Pyridines are a class of synthetic and naturally occurring heterocyclic compounds with a six-membered heterocyclic moiety that has a wide variety of biological and therapeutic applications, including anticancer activities [4,5,6,7]. Recently, we succeeded in synthesizing novel 2-amino-4-aryl-6-substituted pyridine-3,5-dicarbonitrile derivatives using a facile one-pot multicomponent condensation reaction. All the synthesized pyridine derivatives exhibited promising in vitro cytotoxic activities against various cancer cell lines, which grants their subsequent use in cancer therapy [7]. Nevertheless, these compounds showed limited aqueous solubility that might limit their clinical use. Furthermore, the lack of target selectivity of these synthesized pyridine derivatives might compromise their therapeutic effectiveness in vivo.

Recently, the application of nanotechnology has enabled a tremendous push to enhance the therapeutic efficacies of many anticancer agents. Nanoparticulate delivery systems have offered many advantages over conventional delivery systems, including protecting the drug from being degraded/eliminated during its transit to the target site, modifying the biodistribution of the entrapped therapeutic agents, promoting targeted delivery of high drug payloads, controlling drug release, and evading multidrug resistance mechanisms [8,9,10]. Accordingly, a number of nanoparticulate drug delivery systems with an average size below 200 nm, such as micelles [11], liposomes [12], lipid/polymeric nanoparticles [13], dendrimers [14], etc., has been screened for their possible application in cancer therapy. Among these carriers, nanocapsules (NC) have emerged as potential nanocarriers in cancer therapy. Nanocapsules are vesicular systems with a typical core–shell structure that confines active molecules as a reservoir in a cavity surrounded by a polymer coating [15]. The cavity can hold an active material in solid, liquid, or molecular dispersion form [16,17].

Lipid nanocapsules (LNCs) are considered a hybrid structure between liposomes and polymeric nanocapsules owing to their oily core, which is surrounded by a tension-active rigid membrane [18]. Nevertheless, compared to liposomes, LNCs offer the advantages of being of higher physical stability, higher gastrointestinal stability, and having a higher encapsulation capacity of lipophilic drugs in their oily core [18,19]. Most importantly, LNCs show adjuvant effects, such as P-glycoprotein inhibition properties, which could promote preferential intracellular drug accumulation within cancer cells, resulting in enhanced anticancer effects [20]. Recently, many reports have established the in vivo effectiveness of methotrexate-loaded lipid core nanocapsules [21] and indomethacin-loaded lipid-core nanocapsules [22] in treating glioblastoma, since lipid-core nanocapsules act as a drug shuttle that cross the blood brain barrier to deliver the drug to brain tissue.

The aim of this study was to investigate the efficiency of LPNCs in enhancing the anticancer activity of novel pyridine derivatives. LPNCs, composed of polylactic-co-glycolic acid (PLGA) with one of different oil phases (caprylic/capric triglyceride, olive oil, or oleic acid), were prepared by a nanoprecipitation method and were characterized in terms of morphology and physicochemical properties. The anti-proliferative activities of different pyridine derivatives entrapped within LPNCs were investigated in vitro against A549 and MCF-7 cell lines, and in an in vivo tumor model.

## 2. Materials and Methods

### 2.1. Materials

Caprylic/capric triglyceride was obtained from the Gattefosse company (St. Priest, France). 3-(4,5-dimethylthiazol-2-yl)-2,5-diphenyl tetrazolium bromide (MTT) reagent, 5-fluorouracil, poly(D,L-lactide-co-glycolide) lactide:glycolide (PLGA 50:50, Mw 45,000 g/mol), oleic acid, olive oil, and dialysis tubing cellulose membrane (M.Wt. cutoff 12–14 kDa) were obtained from Sigma-Aldrich (St. Louis, CA, USA). All other reagents and chemicals were of analytical grade.

### 2.2. Synthesis Scheme for 2-Amino-4-aryl-6-substituted Pyridine-3,5-dicarbonitrile Derivatives

**Scheme 1:**
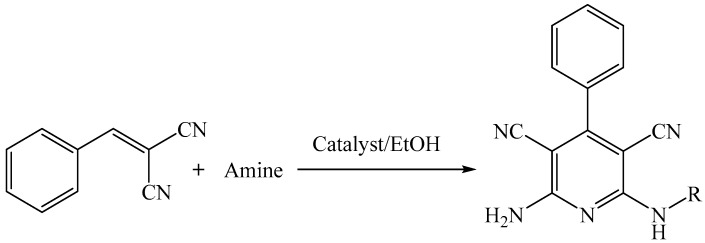
Synthesis scheme for 2-amino-4-aryl-6-substituted pyridine-3,5-dicarbonitrile derivatives.

2-amino-4-aryl-6-substituted pyridine-3,5-dicarbonitrile derivatives were synthesized by a one-pot multicomponent reaction as previously reported [7] (Scheme 1 and Table 1). The structure of the target compounds was completely identified by IR spectra, mass spectra, and ^1^H-NMR spectra as previously shown [7].

### 2.3. Solubility Measurement

The solubility of 2-amino-4-aryl-6-substituted pyridine-3,5-dicarbonitrile derivatives (S1–S4) in various solvents (methanol, ethanol, phosphate buffer, and water) was determined by adding an excess amount of each compound to 10 mL of each solvent in a sealed conical flask. The slurry was then agitated at 100 rpm in a temperature-controlled incubator shaker for 72 h to reach equilibrium. The undissolved particles were allowed to settle by keeping the saturated solution without agitation for another 2 h. The solution was then centrifuged at 10,000 rpm for 10 min. One mL samples of the clear supernatant was withdrawn, diluted with the suitable solvent, and finally analyzed spectrophotometrically at pre-determined wavelengths (Table S1).

### 2.4. Preparation of Lipid-Polymer Nanocapsules (LPNCs)

Lipid-polymer nanocapsules were fabricated by a nanoprecipitation technique [23]. Initially, LPNCs were prepared by an interfacial deposition of polymer. Briefly, a definite weight of PLGA polymer was dissolved in 5 mL of acetone containing 0.25% *w*/*v* Span 60. Then, 1 mM of different pyridine derivatives (S1–S4) previously solubilized in a definite amount of caprylic/capric triglyceride (CCT), oleic acid (OA), or olive oil (VO) was added to the acetonic solution. The resulting organic solution was injected into 10 mL of aqueous solution containing 0.25% *w*/*v* poloxamer 188 or Tween80 under magnetic stirring (Table 2). The mixture was maintained under moderate magnetic stirring overnight to allow the evaporation of the organic solvents and subsequent formation of LPNC. Blank LPNCs were prepared similarly, but without the addition of pyridine derivatives to oil.

### 2.5. Physicochemical Characterization of LPNCs

#### 2.5.1. Particle Size, Polydispersity Index, and Zeta Potential

The particle size and polydispersity index (PdI) of different LPNC dispersions were measured by dynamic light scattering (Nano ZS Zetasizer, Malvern Instruments Ltd., Worcestershire, UK) at 25 °C. Volume-weighted mean diameters (*D*[4,3]) and polydispersity (expressed via measuring the distribution width, span) were estimated by the Malvern software based on the following equations:

$$D\left[4,3\right]=\frac{\sum n_{i}D_{i}^{4}}{\sum n_{i}D_{i}^{3}}$$

$$Span=\frac{D_{v0.9}-D_{v0.1}}{D_{v0.5}}$$

where *I* is an index of the population, *D_i_* is the particle diameter of the population *i*, and *D_v_*_0.1_, *D_v_*_0.5_, and *D_v_*_0.9_ are the diameters at the percentiles 10, 50, and 90 under the cumulative size-distribution curve based on the volume of particles. For particle size distribution, test samples were diluted 1:500 *v*/*v* with deionized water before the measurement.

The zeta potential of different LPNC dispersions was measured by electrophoretic mobility and analyzed with the Malvern Nano ZS Zetasizer. All experiments were performed in triplicates.

#### 2.5.2. Entrapment Efficiency Percentage

Drug loading (*DL*%) and entrapment efficiency (*EE*%) of different pyridine derivative-loaded LPNCs were determined indirectly by estimating the concentration of the free compound. Briefly, 1 mL of test compound-loaded *LPNCs* was centrifuged at 15,000 rpm for 30 min. After centrifugation, the concentration of free compounds in the supernatant was estimated spectrophotometrically and the *DL*% and *EE*% were calculated using the following equations:

$$DL\%=\frac{Total\;drug-Free\;drug}{Total\;weight\;of\;LPNCs}\times 100$$

$$EE\%=\frac{Total\;drug-Free\;drug}{Total\;drug}$$

#### 2.5.3. Surface Morphology

The surface morphology of the prepared LPNCs was examined using a transmission electron microscopy (JEOL JEM 1010, JEOL USA, Inc., Peabody, MA, USA). Briefly, a drop of LPNC dispersion was applied to a copper grid coating, and a filter paper was used to remove the excess droplets. After 5 min, a drop of 2% *w*/*v* uranyl acetate solution was then put onto the grids. After the samples were negatively stained and air-dried at room temperature, they were imaged by TEM at an acceleration voltage of 74 kV.

#### 2.5.4. Differential Scanning Calorimetry (DSC)

Differential Scanning Calorimetry (DSC) was used to study the physical state, crystallinity of each compound, and possible physical interactions of it with the solid excipient to be used in LPNC formulations. The measurement was performed with a differential scanning calorimeter (Shimadzu DSC-60, Tokyo, Japan). Samples weighing 3–7 mg were sealed in aluminum pans and a heating rate of 10 °C/min was employed in a temperature range of 25 to 300 °C under nitrogen gas flow (30 mL·min^−1^). An empty aluminum pan was used as a reference in the study.

### 2.6. In Vitro Release Studies

The in vitro release of each compound was assessed by the dialysis bag method as described previously [24]. Briefly, 1 mL of the LPNC formulation of each compound was tightly sealed in a dialysis tubing (MW cutoff 12−14 kDa) and immersed in 30 mL of release medium (phosphate buffer saline, pH 7.4) kept at 37 °C and stirred at a constant stirring speed (100 rpm). At scheduled time intervals (0.5, 1, 2, 4, 6, 8, 12, 24, 48, 72, 96, 120, 144, and 168 h), 1 mL samples were collected and replaced by an equal volume of fresh release medium. The concentration of various pyridine derivatives in each sample was then quantified spectrophotometrically at specified wavelengths.

### 2.7. In Vitro Cytotoxicity Assay of Pyridine Derivatives-Loaded LPNCs

A human lung cancer (A549) cell line, human breast adenocarcinoma (MCF-7), and normal MCF-10A human breast cancer cells were obtained from VASCERA Co. (Vaccines Sera and Drugs; Cairo, Egypt). Both cell lines were cultured in Dulbecco’s modified Eagle’s medium (DMEM, Biowest, Nuaillé, France) supplemented with 10% fetal bovine serum, 100 U/mL penicillin/100 μg/mL streptomycin, and incubated under standard conditions (37 °C, 5% CO_2_).

The in vitro cytotoxicities of various pyridine derivatives loaded into LPNCs against A549 cells and MCF-7 cells were evaluated by the MTT assay [25]. Briefly, 100 μL cell suspension equivalent to 1 × 10^4^ cells/well was planted in a 96-well plate and incubated at 37 °C. At 24 h post-incubation, the cells were treated with 100 μL of serial dilutions of either free pyridine derivatives (S1–S4) or pyridine derivatives (S1–S4)-loaded LPNCs in a concentration range of 0.1 to 100 μM. The cells were further incubated at 37 °C for 48 h. The spent culture medium was then decanted, the cells were rinsed, 50 μL of MTT reagent (0.5 mg/mL) was added to each well, and the cells were incubated for 4 h at 37 °C. Finally, to dissolve the formazan crystals, DMSO solution (150 μL) was added to each well. The absorbance of each well was measured at 570 nm using a microplate reader (TEKAN Japan, Kawasaki, Japan). The percent cell viability was plotted against the tested compound concentration to determine the IC_50_ values.

### 2.8. In Vivo Studies

#### 2.8.1. Animals

Female Swiss Albino male mice (20–25 g, 6–8 weeks) were provided by the animal house Faculty of Pharmacy in Suez Canal University and kept under specific pathogen-free conditions with free access to standard water and food. All animal studies were reviewed and approved by the Faculty of Pharmacy Ethical Committee Acts (201611PHDA2) of Suez Canal University, Ismailia, Egypt.

#### 2.8.2. Median Lethal Dose (LD_50_) of Test Selective Compound

The median lethal dose (*LD*_50_) test was performed to determine the dose of a test substance that elicits 50% death in mice [26]. Briefly, a total of 30 mice were categorized into 6 groups (n = 5). Each group was dosed with a single intraperitoneal injection of the test compound (S4) dissolved in 3% dimethyl sulfoxide. The screened doses ranged from 100 to 180 mg/kg. The animals were then monitored for 2 h and then at 4, 6, and 24 h for signs of toxicity. At the end of 24 h, the total number of deaths was recorded. The *LD*_50_ was then calculated using the following equation [27]:

$${LD}_{50}={LD}_{100}-\frac{\left\{\Sigma [Dose\;difference\times Mean\;dead]\right\}}{Number\;of\;animals\;per\;group}$$

where *LD*_100_ is the lowest dose capable of killing 100% of animals.

#### 2.8.3. Acute Toxicity Study

Acute toxicity studies are carried out in order to evaluate the short-term negative effects of a test compound when administered in a single dose during a period of 24 h. Briefly, the maximum tolerated dose (100 mg/kg) of the test compound (S4), dissolved in 3% dimethyl sulfoxide, was injected intraperitoneally in a group of mice (n = 5). A control group (n = 5) was i.p. injected with an equivalent volume of 3% dimethyl sulfoxide. The mice were then observed for general behavior for 1 h post-treatment and then intermittently for 4 h and finally at 24 h. The mice were then monitored for up to 14 days after treatment for symptoms of toxicity or mortalities.

#### 2.8.4. Induction of Solid Tumor in Mice

Ehrlich ascites carcinoma (EAC) cells (1 × 10^6^ cells) obtained from the National Cancer Institute (Cairo, Egypt) were injected intraperitoneally into the peritoneal cavity of Swiss Albino mice. On day 7 post tumor cell inoculation, ascites fluid was collected. EAC was diluted with saline to obtain a cell density of 25 × 10^6^ cells/mL. Then, 200 μL of the cells were injected subcutaneously in the mice’s right flanks. Tumor growth was monitored post-inoculation, and the size of the tumor was measured with a Vernier caliper. Treatment protocols were initiated on day 8 post tumor inoculation, when the tumor was palpable.

#### 2.8.5. Treatment Protocol

One week after tumor implantation, when the tumor was palpable, the mice were randomly categorized into five groups (n = 8). All treatments were initiated on day 8 and extended to day 29 post implantation (a three-week treatment period).

Group 1 (EAC control group): EAC-bearing mice were i.p. injected with 0.4 mL saline once every other day for a period of 21 days.
Group 2: EAC-bearing mice were i.p. injected with blank LPNCs once every other day for a period of 21 days.
Group 3: EAC-bearing mice were i.p. injected the standard anticancer drug (5-fluorouracil; 5-FU) at a dose of 10 mg/kg [28] every other day for a period of 21 days.
Group 4: EAC-bearing mice were i.p. injected with free test compound(S4) at a dose of 10 mg/kg) once every other day for a period of 21 days.
Group 5: EAC-bearing mice were i.p. injected with test compound (S4)-loaded LPNCs at a dose of 10 mg/kg) once every other day for a period of 21 days.

The dose and route of administration of the test compound and the treatment regimen were established based on previously published reports [29,30]. The antitumor efficacy of different treatment protocols was evaluated in terms of tumor size, percentage tumor growth inhibition (% TGI), median survival time (MST), and percentage increase in life span (% *ILS*) using the following equations [31,32]:

Tumor size (mm^3^) = ½ (Length × Width^2^)

$$\%\;\mathrm{TGI}=\left[1-\frac{Mean\;tumor\;volume\;of\;treated\;group}{Mean\;tumor\;volume\;in\;control\;group}\right]\times 100$$

*MST* = (day of first death + day of last death)/2

$$\%ILS=\left[\frac{{MST}_{treated\;group}}{{MST}_{control\;group}}-1\right]\times 100$$

In addition, body weight was assessed concurrently to determine any remarkable systemic toxicity.

#### 2.8.6. Histopathological Examination of Solid Tumors

On the last day of the experiment, solid tumors were dissected from all mice and were fixed overnight in formalin-alcohol for 48 h, dehydrated in an alcohol series, cleared in xylene, and embedded in paraffin. Sections (4–5 μm in thickness) were stained with hematoxylin and eosin (H&E). Slides were then imaged using a Universal Infinity System optical microscope (Olympus^®^, Tokyo, Japan) equipped with a computer-controlled digital camera [33]. A histopathological analysis of the tumor sections was focused on finding a typical mitotic picture, neoplastic giant cells, and the extension of the necrotic area. Histopathological examinations were evaluated in a blinded way by a pathologist.

#### 2.8.7. Biochemical Analysis for Liver and Kidney Functions

On the last day of the experiment, 4 out 8 mice were euthanized, and blood samples were collected by cardiac puncture in dry Eppendorf tubes. Blood samples were set aside for 30 min at room temperature and the tubes were then centrifuged for 15 min at 2000× *g* to obtain serum. To assess possible hepatic damage, aspartate transaminase (AST) and alanine transaminase (ALT) activities in serum samples were assayed by using a commercial kit (Biodiagnostic, Cairo, Egypt) according to the method of Reitman and Frankel [34]. In addition, serum creatinine level was quantified as an indicator for possible renal damage. The level of serum creatinine was analyzed by using a commercial kit that was purchased from Biodiagnostic (Cairo, Egypt) according to manufacturer instructions.

### 2.9. Statistical Analysis

A statistical analysis was performed using Graph Pad Prism version 9 by one-way ANOVA followed by a post-hoc Bonferroni’s comparison test. A value of *p* < 0.05 was considered significant. All data were expressed as mean ± standard deviation.

## 3. Results and Discussion

### 3.1. Solubility Measurement of Various Pyridine Derivatives (S1–S4) in Different Solvents

The aqueous solubility of a drug is a fundamental property that plays a crucial role in drug effectiveness. Accordingly, solubility studies of various pyridine derivatives (S1–S4) were conducted in different solvents, namely, water, ethanol, methanol, and phosphate buffer (pH 7.4). As shown in Table S2, the maximum solubility of all the tested compounds (S1–S4) was observed in organic solvents, ethanol, and methanol. On the other hand, all the tested compounds showed poor solubility in both water and the phosphate buffer. These results imply that the solubility of test compounds decreases with the increasing polarity of the solvents. Most importantly, the poor aqueous solubility of test compounds might limit their therapeutic efficacy since poor aqueous solubility would be associated with poor drug bioavailability. Consequently, in order to overcome such limitations, the test compounds were loaded into lipid polymer nanocapsule (LPNC) formulations and investigated for their anticancer potential both in vitro and in vivo.

### 3.2. Preparation of Lipid Polymer Nanocapsules (LPNCs)

During the development process, a variety of materials and their combinations were adopted for the preparation of LPNCs (Table 2). First, PLGA, at variable amounts, was employed as the shell-forming polymer, while caprylic/capric triglyceride (CCT), oleic acid, or olive oil, at variable amounts, were tested as the main oily component of the developing LPNC formulations. The shell-forming polymer (PLGA) was dissolved in acetone in the presence of two different surfactants (poloxamer 188 or Tween 80). All the developed formulations were screened for particle size. The formulation that showed the smallest particle size was selected as the optimized formula.

#### 3.2.1. Effect of Polymer Content and Oil Content on the Size Distribution of LPNCs

Polymer and oil contents are considered the most important factors influencing the structure of LPNC formulations [35]. Accordingly, in this study, the impact of both polymer and oil content on the size distribution of the formulated LPNCs was investigated. LPNCs were prepared using variable amounts of PLGA (10, 20, and 30 mg) and variable amounts of CCT (100, 150, and 200 mg) in the presence of poloxamer 188 as a surfactant. The particle size distribution of the prepared LPNCs, in terms of volume mean diameter (*D*[4,3]) and polydispersity index (PDI), is summarized in Table 3 (F1–F5). The laser diffraction analysis confirmed the presence of all formulated LPNCs in the nano-size range with a mean diameter (*D*[4,3]) ranging from of 135.33 ± 0.58 to 225.00 ± 10.54 nm and with a span value fluctuating from 0.99 ± 0.01 to 1.82 ± 0.23. Generally, the span is used to quantify distribution width; span values below 2.0 indicate narrow particle size distribution for these formulations. Of note, at fixed PLGA contents the mean diameter (*D*[4,3]) of different LPNC formylations was found to increase gradually from 135.33 ± 0.58 to 225.00 ± 10.54 nm with increasing oil content from 100 to 200 mg. Such an increase in particle size with rising CCT content might be ascribed to the high viscosity of the organic phase, since the higher the oil content, the more viscous the organic phase [36]. Furthermore, it was evident that at fixed oil content, increasing polymer content from 10 to 30 mg triggered a mutual rise in the mean diameter (*D*[4,3]) of LPNCs. The mean diameter (*D*[4,3]) of F1 prepared with 10 mg PLGA showed a mean diameter (*D*[4,3]) of 135.33 ± 0.58 nm, which is remarkably smaller than that of LPNCs prepared with 30 mg PLGA (F5 184.67 ± 6.43 nm). Nevertheless, increasing the polymer content from 10 to 20 mg did not result in a remarkable change in the mean diameter of formulated LPNCs. Apart from its effect on particle size, it is well-recognized that the polymeric shell plays a key role in protecting encapsulated drugs and maintaining formulation stability. Accordingly, the stability of LPNCs prepared with 10 and 20 mg PLGA was investigated. Two weeks after preparation, a remarkable increase in particle size of LPNCs prepared with 10 mg PLGA from 135.33 ± 0.58 to 146.33 ± 3.21 nm was observed, while no remarkable increase in particle size was detected in LPNCs prepared with 20 mg PLGA. Based on these findings, a polymer content of 20 mg and an oil content of 100 mg was selected for the formulation of LPNCs for further experiments.

#### 3.2.2. Effect of Oil Type and Surfactant Type on the Size Distribution of LPNCs

Next, we investigated the effect of oil type on the size distribution of LPNCs. In this set of experiments, the size distribution of LPNCs prepared with 100 mg oleic acid or olive oil was estimated and compared with that of LNCPs prepared with CCT. As depicted in Table 3, the type of oil significantly affected the mean diameter (*D*[4,3]) of different LPNC formulations. The mean diameter (*D*[4,3]) of LPNCs prepared with different oils was in the following order: CTT (136.00 ± 1.73 nm) < oleic acid (255.00 ± 40.63 nm) < olive oil (346.67 ± 26.03 nm). Viscosity regulates the mobility of the organic phase into the aqueous phase, and thus directly affects the emulsification and nanoparticles size [36]. This might explain the smaller size of LPNCs prepared with the low viscous oil (CCT) compared to the higher viscous oils (oleic acid and olive oils).

Many reports have emphasized the contribution of the hydrophilic lipophilic balance (HLB) of the used surfactants on particle size and formulation stability. In this study, however, it was evident that surfactant types exerted no distinct effect on the particle size of the formulated LPNCs (Table 2). LPNCs prepared with 20 mg of PLGA, 100 mg of CCT, and 0.25% *w*/*v* Poloxamer 188 (F4) showed the smallest particle size amongst all tested formulations; consequently, this formulation was selected as an optimized formula for encapsulating different pyridine derivatives (S1–S4).

### 3.3. Preparation of Each Compound-Loaded LPNC

Based on formulation development data, PLGA (20 mg) as a shell-forming polymer, CCT (100 mg) as the oily core, and poloxamer 188 (0.25% *w*/*v*) as the stabilizer were selected as the components for the fabrication of pyridine derivatives (S1–S4)-loaded LPNCs. Briefly, 1 mM of the test compounds were initially solubilized in CCT, and then the oily phase was added to polymer acetonic solution. Finally, the resulting organic solution was injected into 10 mL of water containing 0.25% *w*/*v* poloxamer 188 under magnetic stirring. The formulated test compound-loaded LPNCs were then characterized for mean particle size and size distribution, surface charge, and morphology.

### 3.4. Characterization of Test Compound-Loaded LPNCs

#### 3.4.1. Particle Size and Size Distribution

Particle size plays a crucial role in dictating the therapeutic efficacy of drug-loaded nanocarriers. Generally, most nanocarrier systems used for cancer therapy are designed to be between 50 to 200 nm to favor the enhanced permeation and retention (EPR) effect [37]. As listed in Table 4 and Figure S1, various test compound-loaded LPNCs had a particle size ranging from 185.0 ± 17.4 (S1-loaded LPNCs) to 223.0 ± 15.3 nm (S4-loaded LPNCs). This size range of all formulated LPNCs is large enough (>50 nm) to evade hepatic sequestration [38], while it is small enough to escape phagocytosis (<250 nm) [39]. Of note, the polydispersity index (PDI) of all formulations is <0.3, indicating narrow size distribution [40].

#### 3.4.2. Zeta Potential of Test Compound-Loaded LPNCs

The zeta potential in another important parameter that contributes to the stability of colloidal dispersions. Generally, colloidal dispersions having a high zeta potential value of ±20 mV are believed to have good dispersion stability and a low chance of aggregation [41]. As summarized in Table 4 and Figure S1, the zeta potentials of all LPNC formulations were in the negative range, fluctuating from −7.55 ± 1.7 (S2-LPNCs) to −13.4 ± 2.1 mV (S4-LPNCs). The negative zeta potential of all formulations might be ascribed to the presence of the terminal carboxylic acid group in PLGA used as a shell-forming polymer [42]. This negative zeta potential might contribute to the colloidal stability of formulated LPNCs via hindering particle aggregation and/or precipitation by virtue of electrostatic repulsion between negatively charged surfaces.

#### 3.4.3. Morphology of Test Compound-Loaded LPNCs

**Figure 1:**
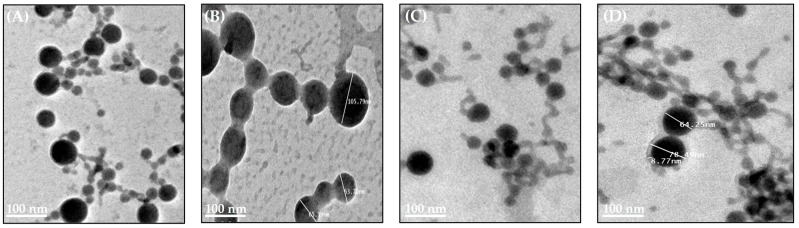
TEM micrograph depicting the shape and morphology of (**A**) S1-loaded LPNCs; (**B**) S2-loaded LPNCs; (**C**) S3-loaded LPNCs; and (**D**) S4-loaded LNCPs. The scale bar equals 100 nm.

In order to investigate the morphology of LPNCs, transmission electron microscopy (TEM) analysis was conducted (Figure 1). As shown in Figure 1, all the formulated LPNCs were spherical in shape, having smooth surfaces. Of note, TEM photographs showed that the particle size of the nanoparticles was around 100–200 nm. Small variations in particle size estimated by TEM, compared to the DLS technique, might be ascribed to differences in measurement conditions. In TEM imaging, the size of nanocapsules is determined in a dry state, while in DLS, the nanoparticles are in a hydrated state, where each particle is surrounded by a solvent sheath. This could explain the larger size of LPNCs estimated by DLS compared to that determined by TEM analysis [43].

#### 3.4.4. Drug Loading and Entrapment Efficiency of Test Compound-Loaded LPNCs

Efficient drug loading is a key determinant of the therapeutic efficacy of drug-loaded nanocarriers. As summarized in Table 4, the drug-loading percentage of different test compounds within LPNCs varied from 1.75 ± 0.03 (for S3-loaded LPNCs) to 2.39 ± 0.01% (for S2-loaded LPNCs), respectively. Regarding encapsulation efficiency, it was evident that the entrapment efficiency of different test compounds within LPNCs fluctuated from 90.62 ± 0.52% (for S2-loaded LPNCs) to 93.72 ± 0.66% (for S4-loaded LPNCs). The significantly high encapsulation efficiency of different pyridine derivatives within LPNCs might be explained by the greater solubility of compounds in the oily component of these formulations. Similar results were reported by de Melo et al. [44], who emphasized the impact of the composition of the oily nucleus on the encapsulation of the hydrophobic drug benzocaine within PLGA nanocapsules.

#### 3.4.5. Differential Scanning Calorimetry Analysis

**Figure 2:**
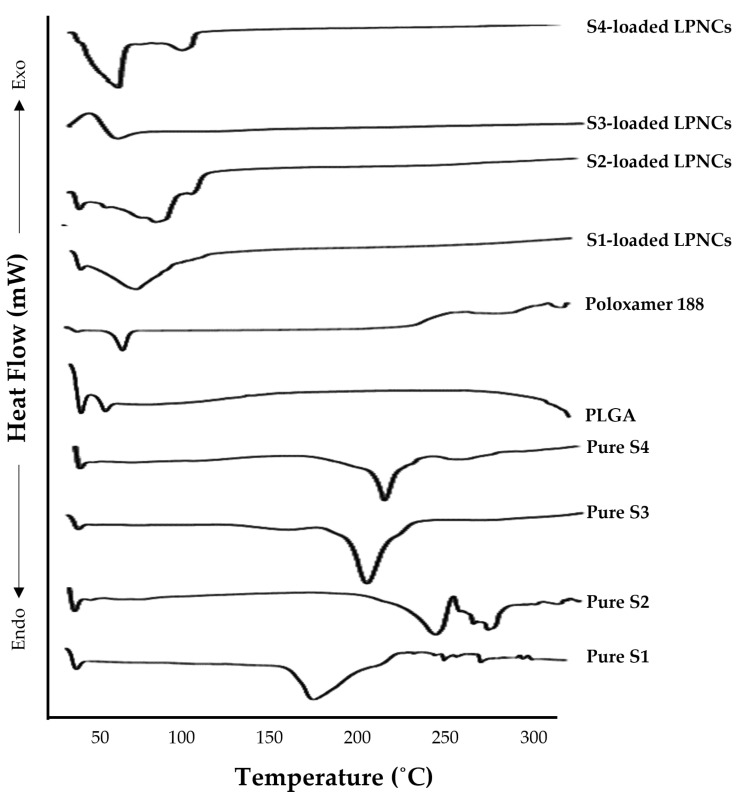
DSC thermograms of free test compounds (S1–S4); PLGA; Poloxamer 188; and various test compounds (S1–S4)-loaded LPNC formulations.

DSC is an important technique for analyzing the crystallinity and interaction of drugs with different components of nanocapsules by determining the alteration of temperature and energy at phase transition. In order to investigate the effect of the formulation process on the thermal behavior of the formulations, a DSC analysis of each test compound in pure form, PLGA, poloxamer 188, and different LPNC formulations was conducted (Figure 2). DSC thermograms of the pure substances (S1, S2, S3, and S4) showed characteristic sharp peaks at 167.13 °C, 224.98 °C, 186.18 °C, and 194.38 °C, respectively, indicating the crystalline nature of the test compounds. The PLGA thermogram showed a sharp endothermic melting peak at 47.33 °C, which might correspond to the glass transition temperature of PLGA [45]. Similarly, the Poloxamer 188 thermogram showed the sharp endothermic melting peak at 54 °C, which corresponds to the melting peak of Poloxamer 188. Of interest, DSC thermograms of test compound-loaded LPNCs did not show the melting peaks of pure compounds, suggesting that each compound was either converted into an amorphous form or was molecularly dispersed in the formulated LPNCs. Furthermore, the DSC study indicated the absence of any drug-excipient incompatibility between the compounds and excipients.

### 3.5. In Vitro Release of Test Compounds from LPNCs

**Figure 3:**
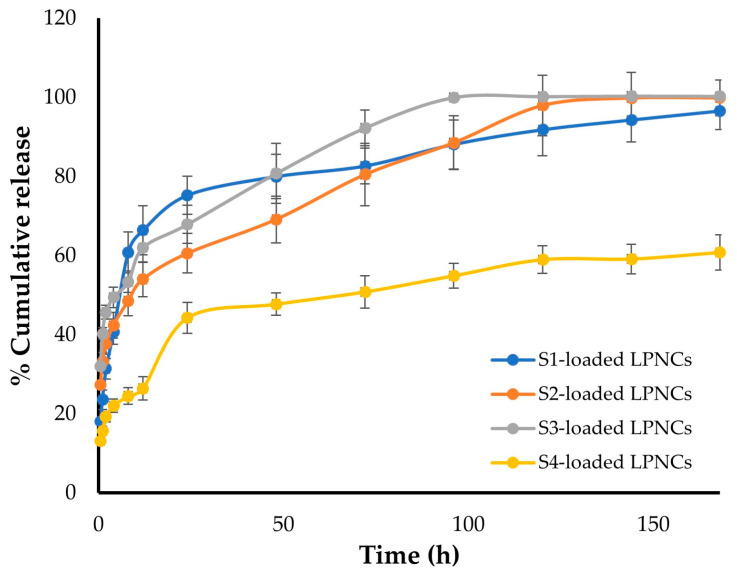
In vitro release profile of test compounds (S1–S4) from test compound-loaded LPNCs. Values are expressed as mean ± SD (n = 3).

The release profile of various test compounds from LPNC formulations was assessed for 7 days and the cumulative drug release was plotted as a function of time (Figure 3). As shown in Figure 3, all test compound-loaded LPNC formulations showed a biphasic release pattern with an initial burst release of compounds in the first 12 h, followed by a sustained drug release for up to 168 h. The percent cumulative drug release in the first 12 h was 66.4 ± 3.2%, 54.0 ± 4.4%, 61.9 ± 3.7%, and 26.4 ± 2.9% for S1-, S2-, S3-, and S4-loaded LPNCs, respectively. This biphasic release pattern might be attributed to the core–shell structure of lipid-polymer nanocapsules, in which the compounds could be associated with the surface of the nanocapsules and/or dissolved into the core [44]. Of note, among different LPNC formulations, S4-loaded LPNCs showed the lowest burst release of compound S4, suggesting the efficient entrapment of compound S4 in the oily core rather than being adsorbed at the outer surface of the nanocapsule polymeric shell. Furthermore, like other formulations, LPNCs could sustain the release of compound S4 for up to 7 days. Such a release pattern would be favored in cancer therapy, where a sustained drug release would grant the efficient delivery of an adequate concentration of the entrapped drug to the target tissue for a minimum of 4–7 days post-formulation-administration, which in turn would enhance patient compliance.

### 3.6. In Vitro Cytotoxicity Assay of Test Compound-Loaded LPNCs

In order to address the possible potentiating effect of test compound encapsulation within LPNCs on the therapeutic efficacy of different pyridine derivatives, the in vitro cytotoxicities of different test compounds (S1–S4) and test compound-loaded nanocapsules were investigated against both the breast cancer MCF 7 cell line and the lung cancer A549 cell line at concentrations (0.1, 1, 10, and 100 μM) using MTT assay. Both cancer cell lines were selected based on our previous results [7] depicting the weak cytotoxic potential of free test compounds against both cell lines. The IC_50_ values of different formulations against MCF-7 and A549 cell lines are summarized in Table 5. The results reported in Table 5 clearly indicate that the test compound-loaded LPNCs were more cytotoxic against both cell lines compared to the free counterparts. All tested free compounds exerted IC_50_ values of >100 μM against either MCF-7 or A549 cells. On the other hand, test compound-loaded LPNCs significantly augmented the cytotoxic potential of entrapped drugs as manifested by a significant decrease in the IC_50_ values. The IC_50_ values against MCF-7 cells were 21.9 ± 1.3, 28.3 ± 1.7, 28.2 ± 1.4, and 9.33 ± 0.9 μM for S1-, S2-, S3-, and S4-loaded LPNCs, respectively. Similarly, the IC_50_ values against A549 cells were 63.1 ± 3.2, 51.3 ± 2.9, 58.9 ± 2.6, and 28.8 ± 1.1 μM for S1-, S2-, S3-, and S4-loaded LPNCs, respectively. Of note, blank LPNCs did not exert any significant cytotoxic effect against either tested cancer cell line at any of the tested concentration ranges. Consequently, the superior cytotoxic activity of different test compound-loaded LPNCs, compared to free compounds, might be accounted for by the enhanced cellular uptake/internalization of nanocapsules by cancer cells [46]. Of note, among different drug-loaded nanocapsule formulations, S4-loaded LPNCs showed the highest cytotoxic potential against both tested cancer cell lines. The relatively slower release of S4 from LPNCs compared to other formulations might account for the superior cytotoxic activities of S4-loaded LPNCs against both cancer cell lines. Similar results were reported by Sethi et al. [47], who attributed the enhanced in vitro and in vivo cytotoxic potential of docetaxel-loaded nanoparticles to the sustained drug release pattern from nanoparticles. Based on our in vitro cytotoxicity results, S4-loaded LPNCs were selected for further in vivo investigations.

Finally, to rule out the possible cytotoxic potential of S4-loaded LPNCs against normal healthy cells, the cytotoxicity of S4-loaded LPNCs was screened against noncancerous, normal MCF-10A human breast cancer cells, and the selectivity index was calculated. As shown in Figure S2, no remarkable decrease in the viability of MCF-10A cells was detected at test compound concentrations up to 100 μM. However, at relatively higher concentrations (200 and 400 μM), S4-loaded LPNCs could induce an obvious cytotoxic effect against MCF-10A cells. The IC_50_ value of S4-loaded LPNCs cells was 198.5 ± 13.2 μM. Most importantly, the calculated selectivity index, defined as the ratio of the average IC_50_ value against the noncancerous cell line (MCF-10A) to that in the cancer cell line (MCF-7), was found to be 21.3, indicating the great selectivity of S4-loaded LPNCs against cancerous cell lines rather than normal cells.

### 3.7. In Vivo Studies

#### 3.7.1. Median Lethal Dose (LD_50_) of Compound (S4) in Female Albino Mice

Initially, to investigate the appropriate dose of test compound (S4) for in vivo use, mice were intraperitoneally injected with escalating doses of S4 ranging from 100 to 180 mg/kg. The animals were then monitored periodically for 24 h, and the LD_50_ was computed at 24 h post-injection. As summarized in Table S3, high doses of test compound S4 (120 mg/kg and higher) were lethal to animals, while the lower dose (100 mg/kg) was not lethal but only induced drowsiness in test animals, which recovered after 24 h. The calculated LD_50_ of compound (S4) in adult female mice was found to be 50 mg/kg body weight after intraperitoneal injection.

#### 3.7.2. Acute Toxicity

An acute toxicity study was carried out to outline the short-term adverse effects of the maximum tolerated dose (100 mg/kg) of test compound S4. According to the acute toxicity study, no behavioral abnormalities, toxicity symptoms, or mortalities were detected in mice at test compound S4 levels up to 100 mg/kg, suggesting the safety of the test compound at this dose.

#### 3.7.3. Antitumor Activity of S4-Loaded LPNCs

**Figure 4:**
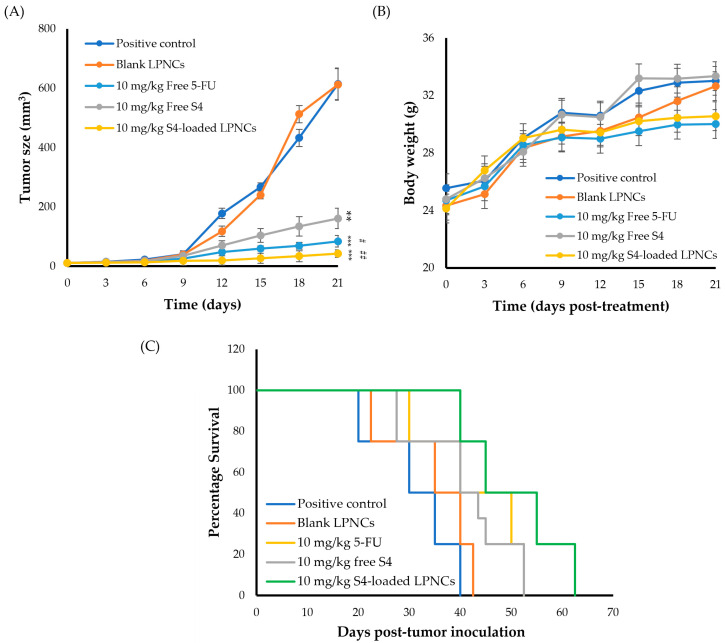
In vivo antitumor efficacy of various S4 formulations on EAC tumor-bearing mice. At days 8 after tumor inoculation, either blank LPNCs, free 5-FU (10 mg/kg), free S4 (10 mg/kg), or S4-loaded LPNCs (10 mg/kg) were i.p. administered. (**A**) Tumor size; (**B**) Survival of EAC-bearing mice; and (**C**) Body weight changes during the treatments. ** *p* < 0.01 and *** *p* < 0.001 vs. positive control, # *p* < 0.05 and ## *p* < 0.01 vs. free S4-treated mice.

In order to address whether the in vitro cytotoxicity results would be reflected by the in vivo therapeutic activity, the in vivo antitumor activity of different test compound (S4) formulations in a subcutaneous EAC tumor-bearing mouse model was assessed. Mice were treated with distilled water, blank nanocapsules, standard chemotherapeutic agent (5-FU), free S4 compound (10 mg/kg), or S4-loaded PLNCs (10 mg S4 per kg) three times a week for three weeks via intraperitoneal injection. The administered dose of S4 was determined based on preliminary studies, which showed the safety and tolerability of this test dose following subsequent administrations. As depicted in Figure 4A, the treatment of mice with standard chemotherapeutic agent (5-FU), free test compound (S4), or S4-loaded LPNCs resulted in a considerable tumor growth inhibitory effect compared with either control (non-treated) mice or blank LPNCs-treated mice. Most importantly, consistent with our in vitro cytotoxicity experiments, S4-loaded LPNCs exerted a superior antitumor efficacy compared to free S4. In addition, when compared with all treated groups, S4-loaded LPNCs strongly suppressed tumor growth in the EAC tumor-bearing mouse model; the tumor growth inhibition rate (TGI (%)) was 92.83%, which was remarkably higher than that of either the standard chemotherapeutic agent (5-FU) or the free S4-treated-group (TGIs (%) were 86.81% and 73.42%, respectively). This enhanced antitumor efficacy of test compound-loaded LPNCs might be ascribed to the efficient entrapment of the test compound within the inner oily core of nanocapsules, which could efficiently protect the entrapped payload from being prematurely released into systemic circulation during its transit to the target tumor tissue, and thereby grant the delivery of an adequate concentration of entrapped drug to the target site.

Furthermore, S4-loaded LPNCs exhibited a pronounced survival advantage, where 50% of the treated mice (four out of eight) became long-term survivors for up to 63 days post-treatment compared to other treated groups (*p* < 0.01) (Figure 4B). In addition, S4-loaded LPNCs triggered a remarkable increase in the life spans of tumor-bearing mice. The percent increase in life span (% ILS) in S4-loaded LPNCS-treated mice was 1.54, which was significantly higher than that in mice treated with either standard 5-FU (% ILS 1.33) or free S4-treated mice (% ILS 1.37). On the other hand, blank LPNCs did not show a survival advantage compared with the control group.

Finally, no body weight loss was detected in any of the treated groups under our experimental conditions (Figure 4C). These results suggest that treatment protocols were well-tolerated by mice and that S4-loaded LPNCs could exert their potent antitumor activity in EAC tumor-bearing mice without causing remarkable toxicity.

#### 3.7.4. Histopathological Examination of Solid Tumors

**Figure 5:**
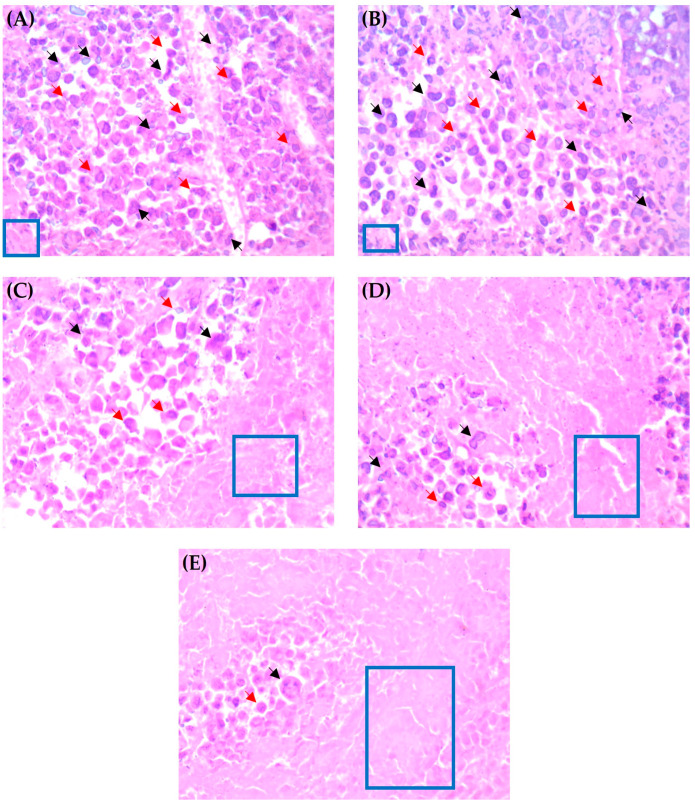
H & E staining of tumor tissue sections collected from (**A**) control group; mice treated with (**B**) blank LPNCs; (**C**) 5-FU (10 mg/kg); (**D**) Free S4 (10 mg/kg); or (**E**) S4-loaded LPNCs (10 mg/kg). Black arrows refer to mitotic figures and red arrows refer to tumor giant cell, while the necrosis area is outlined by the square.

Microscopic examination of H&E-stained tumor sections from the EAC solid tumor of the control and blank LPNC-treated groups (Figure 5) showed typical pathological features of closely arranged tumor cells with clear nuclear structures, numerous bizarre mitotic figures, and giant tumor cells with slight necrosis. On the other hand, tumor sections from animals treated with either 5-FU, free S4, or S4-loaded LPNCs revealed a loss of cellular architecture and tumor tissue compactness as well as the appearance of vacuolar structures within the necrotic region (Figure 5). Of interest, there was a remarkable decrease in the number of mitotic figures and giant cells in S4-loaded LPNC-treated mice compared to either 5-FU-treated or free S4-treated mice (Figure 5). These findings confirm the superior antitumor efficacy of S4-loaded LPNCs compared to either free 5-FU or free S4 compounds.

#### 3.7.5. Biochemical Analysis of Liver and Kidney Functions

The liver and kidney are the two most significant organs in the body for drug metabolism and elimination of drugs in the body, and they are the most susceptible organs to harm by drug-induced effects. Consequently, biochemical assays for serum AST and ALT, as indicators for liver function, and serum creatinine levels, as a marker for kidney function, were performed to ensure the safety of different treatments. Serum levels of different liver and kidney markers post different treatments were recorded in Table 6. Generally, liver injury is associated with elevated levels of ALT and AST [48]. As summarized in Table 5, with the exception of mice treated with 5-FU, no significant alterations in serum levels of either ALT and AST were observed upon treatment with blank LPNCs, free S4, or S4-loaded LPNCs. Similarly, kidney function, monitored via estimating serum creatinine levels, demonstrated a non-significant increase in serum creatinine levels in all treated animals with the exception of 5-FU, which triggered a significant elevation of serum creatinine levels in 5-FU-treated mice when compared with control animals. Consequently, these results signify the safety of S4-loaded LPNCs following systemic administration and nullify the possibility of the induction of liver injury or renal dysfunction.

## 4. Conclusions

In this study, we succeeded in developing LPNCs, composed of a CCT oily core and PLGA shell, for the efficient entrapment of novel pyridine derivatives (S1–S4). The fabricated nanocapsules could efficiently enhance the aqueous solubility of test compounds (S1–S4), and thereby could contribute to the enhancement of the anticancer potential of entrapped compounds compared to their free counterparts. In vitro cytotoxicity studies clearly demonstrated the superior cytotoxic potential of S4-loaded LPNCs against both MCF-7 and A549 cancer cells compared to free S4. Most importantly, in vivo antitumor experiments emphasized the efficacy of S4-loaded LPNCs in defeating tumor growth and prolonging the survival time of tumor-bearing mice compared to the free test compound (S4). This potent antitumor activity might be ascribed to the sustained release of the test compound from LPNCs, which could grant effective delivery of adequate concentrations of the entrapped drug to tumor tissues following systemic administration. Furthermore, treatment with S4-loaded LPNCs could exert their efficient antitumor effect without eliciting any remarkable systemic toxicities. To sum up, test compound-loaded LPNCs might be a promising candidate for cancer therapy due to their high therapeutic efficacy and low adverse off-target effects.

## Figures and Tables

**Table 1:** Chemical structure of various pyridine derivatives (S1–S4).

Compound Code	Amine	R	Chemical Formula	M·wt
S1	CH_3_CH_2_NH_2_	CH_3_CH_2_	C_15_H_13_N_5_	263
S2	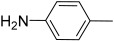	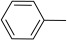	C_20_H_15_N_5_	325
S3	NH_4_OAc	H	C_15_H_9_N_5_	235
S4	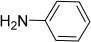	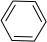	C_19_H_15_N_5_	311

**Table 2:** Composition of various LPNC formulations.

Formula	PLGA (mg)	CCT (mg)	Oleic Acid (mg)	Olive Oil (mg)	Poloxamer 188 (%*w*/*v*)	Tween 80 (%*w*/*v*)
F1	10	100	---	---	0.25	---
F2	10	150	---	---	0.25	---
F3	10	200	---	---	0.25	---
F4	20	100	---	---	0.25	---
F5	30	100	---	---	0.25	---
F6	20	---	100	---	0.25	---
F7	20	---	---	100	0.25	---
F8	20	100	---	---	---	0.25
F9	20	---	100	---	---	0.25
F10	20	---	---	100	---	0.25

PLGA, polylactic-co-glycolic acid (PLGA; 50:50); and CCT, caprylic/capric triglyceride.

**Table 3:** Effect of various formulation variables on particle size distribution of blank LPNCs.

Formula	*Dv*_0.1_	*Dv*_0.5_	*Dv*_0.9_	*D*[4,3]	Span
F1	73.67 ± 0.58	126.67 ± 0.58	210.00 ± 1	135.33 ± 0.58	1.07 ± 0.01
F2	91.00 ± 6.08	172.00 ± 3	326.67 ± 16.07	193.33 ± 2.08	1.37 ± 0.15
F3	80.67 ± 4.62	187.00 ± 9.17	420.67 ± 35.1	225.00 ± 10.54	1.82 ± 0.23
F4	78.67 ± 1.53	128.00 ± 2.65	205.33 ± 2.31	136.00 ± 1.73	0.99 ± 0.01
F5	76.00 ± 3.61	158.00 ± 4.36	334.00 ± 21.63	184.67 ± 6.43	1.63 ± 0.12
F6	86.00 ± 4.36	203.33 ± 10.26	461.67 ± 80.39	255.00 ± 40.63	1.85 ± 0.37
F7	102.33 ± 6.11	253.00 ± 11.53	693.33 ± 73.38	346.67 ± 26.03	2.33 ± 0.17
F8	79.33 ± 4.73	138.67 ± 3.51	238.33 ± 28.31	153.67 ± 11.55	1.14 ± 0.22
F9	80.67 ± 5.03	172.00 ± 9.54	362.67 ± 56.89	201.67 ± 19.73	1.63 ± 0.30
F10	84.33 ± 2.89	201.67 ± 5.77	472.00 ± 22.52	256.00 ± 15.59	1.92 ± 0.19

Data represent mean ± SD of three independent experiments.

**Table 4:** Physicochemical characteristics of various test compound-loaded LPNCs.

Formula	Size Distribution (nm)	PDI	Zeta Potential (mV)	Entrapment Efficiency (%)	Drug Loading (%)
S1-LPNCs	185.0 ± 17.4	0.098 ± 0.01	−7.55 ± 1.7	92.67 ± 0.84	1.99 ± 0.02
S2-LPNCs	187.5 ± 15.2	0.141 ± 0.02	−11.60 ± 1.8	90.62 ± 0.52	2.39 ± 0.01
S3-LPNCs	218.9 ± 10.9	0.200 ± 0.02	−11.90 ± 1.5	91.20 ± 1.70	1.75 ± 0.03
S4-LPNCs	223.0 ± 15.3	0.218 ± 0.03	−13.4 ± 2.1	93.72 ± 0.66	2.37 ± 0.02

Data represent mean ± SD of three independent experiments.

**Table 5:** IC_50_ values of the tested free compounds and test compound-loaded LPNCs against MCF 7 and A549 cell lines.

Formula	IC_50_ (µM) against MCF-7	IC_50_ (µM) against A549
Pure S1	>100	>100
Pure S2	>100	>100
Pure S3	>100	>100
Pure S4	>100	>100
S1-loaded LPNCs	21.9 ± 1.3	63.1 ± 3.2
S2-loaded LPNCs	28.3 ± 1.7	51.3 ± 2.9
S3-loaded LPNCs	28.2 ± 1.4	58.9 ± 2.6
S4-loaded LPNCs	9.33 ± 0.9	28.8 ± 1.1

Data represent mean ± SD of three independent experiments.

**Table 6:** Effects of different S4 formulations on serum levels of blood biochemical parameters.

Groups	AST (IU/L)	ALT (IU/L)	Creatinine (mg/dL)
EAC control	36.06 ± 1.72	17.45 ± 1.31	0.49 ± 0.09
EAC + blank LPNCs	34.61 ± 1.33	16.55 ± 0.71	0.54 ± 0.03
EAC + 5-FU	74.23 ± 3.84 *	51.87 ± 1.29 *	1.05 ± 0.15 *
EAC + Free S4 (10 mg/kg)	36.66 ± 1.87	20.50 ± 1.28	0.54 ± 0.02
EAC + S4-loaded LPNCs (10 mg/kg)	36.13 ± 2.15	18.96 ± 1.56	0.51 ± 0.03

AST = Aspartate aminotransferase; ALT = alanine aminotransferase; EAC = Ehrlich’s ascites carcinoma. Data are exhibited as mean ± SD. * *p* < 0.05 vs. EAC control.

## Data Availability

The data presented in this study are available in this article and Supplementary Materials.

## Supplementary Materials

The following supporting information can be downloaded at: https://www.mdpi.com/article/10.3390/pharmaceutics15061755/s1, Figure S1: (A) Particle size and (B) zeta potential of various test compound-loaded LPNCs; Figure S2: In vitro cytotoxicity of S4-loaded LPNCs against normal noncancerous MCF-10A breast cancer cells; Table S1: Maximum wavelength (λ_max_) of various pyridine derivatives (S1–S4) in acetonitrile, methanol, ethanol, phosphate buffer (pH 7.4), and ethanol:phosphate buffer (1:1); Table S2: Solubility of different pyridine derivatives (S1–S4) in different solvents; Table S3: Results of the lethal doses of compound (S4) for the determination of the LD50 after i.p. injection in female Albino Swiss mice (n = 5).
