# Supplementary material for: In Vitro Cytotoxicity and In Vivo Antitumor Activity of Lipid Nanocapsules Loaded with Novel Pyridine Derivatives
Source: Pharmaceutics. 2023 Jun 16;15(6):1755. doi: 10.3390/pharmaceutics15061755 (PMC10304225; doi:10.3390/pharmaceutics15061755)
Supplement: Supplementary file 1 [file pharmaceutics-15-01755-s001.zip › pharmaceutics-2438010-supplementary.pdf]

pharmaceutics

Supplementary Materials: *In vitro* cytotoxicity and *In vivo* anti-tumor activity of Lipid Nanocapsules Loaded with Novel Pyridine Derivatives

Amr Selim Abu Lila, Mohammed Amran, Mohamed A. Tantawy, Ehssan H. Moglad, Shadeed Gad, Hadil Faris Alotaibi , Ahmad J. Obaidullah, El-Sayed Khafagy

Supplementary Information:

Table S1. Maximum wavelength (λ_{max}) of various pyridine derivative (S1–S4) in acetonitrile, methanol, ethanol, phosphate buffer (pH 7.4), and ethanol:phosphate buffer (1:1).

Solvent	S1		S2		S3		S4	
	Maximum wavelength (λ_{max}) in nm							
	Band 1	Band 2	Band 1	Band 2	Band 1	Band 2	Band 1	Band 2
Acetonitrile	263	323	297	338	258	326	244	335
Methanol	231	330	244	345	270	334	276	330
Ethanol	274	326	237	340	274	329	254	328
Phosphate buffer (pH 7.4)	273	323	232	338	263	327	261	326
Ethanol:Phosphate buffer (1:1)	273	325	238	339	274	329	254	331

Table S2. Solubility of different pyridine derivatives (S1-S4) in different solvents.

	solubility (mg/ml)			
Compound	ethanol	PB (pH 7.4)	water	methanol
S1	5.98 ± 0.230	8.07 ± 0.065	0.57 ± 0.021	21.76 ± 0.260
S2	13.82 ± 0.326	1.63 ± 0.005	1.25 ± 0.006	25.71 ± 0.323
S3	18.86 ± 0.225	0.59 ± 0.010	0.99 ± 0.007	35.36 ± 0.203
S4	18.56 ± 0.337	0.59 ± 0.001	0.58 ± 0.002	16.57 ± 0.315

Table S3. Results of the lethal doses of compound (S4) for the determination of the LD50 after i.p injection in female Albino Swiss mice (n=5).

Groups	Dose mg/kg	Number of animal dead	Dose difference (a)	Mean mortality (b)	Probity (a*b)
1	Control	0	-	-	-
2	100	0	0	-	-
3	120	5	20	2.5	50
4	140	5	20	5	100
5	160	5	20	5	100
6	180	5	20	5	100

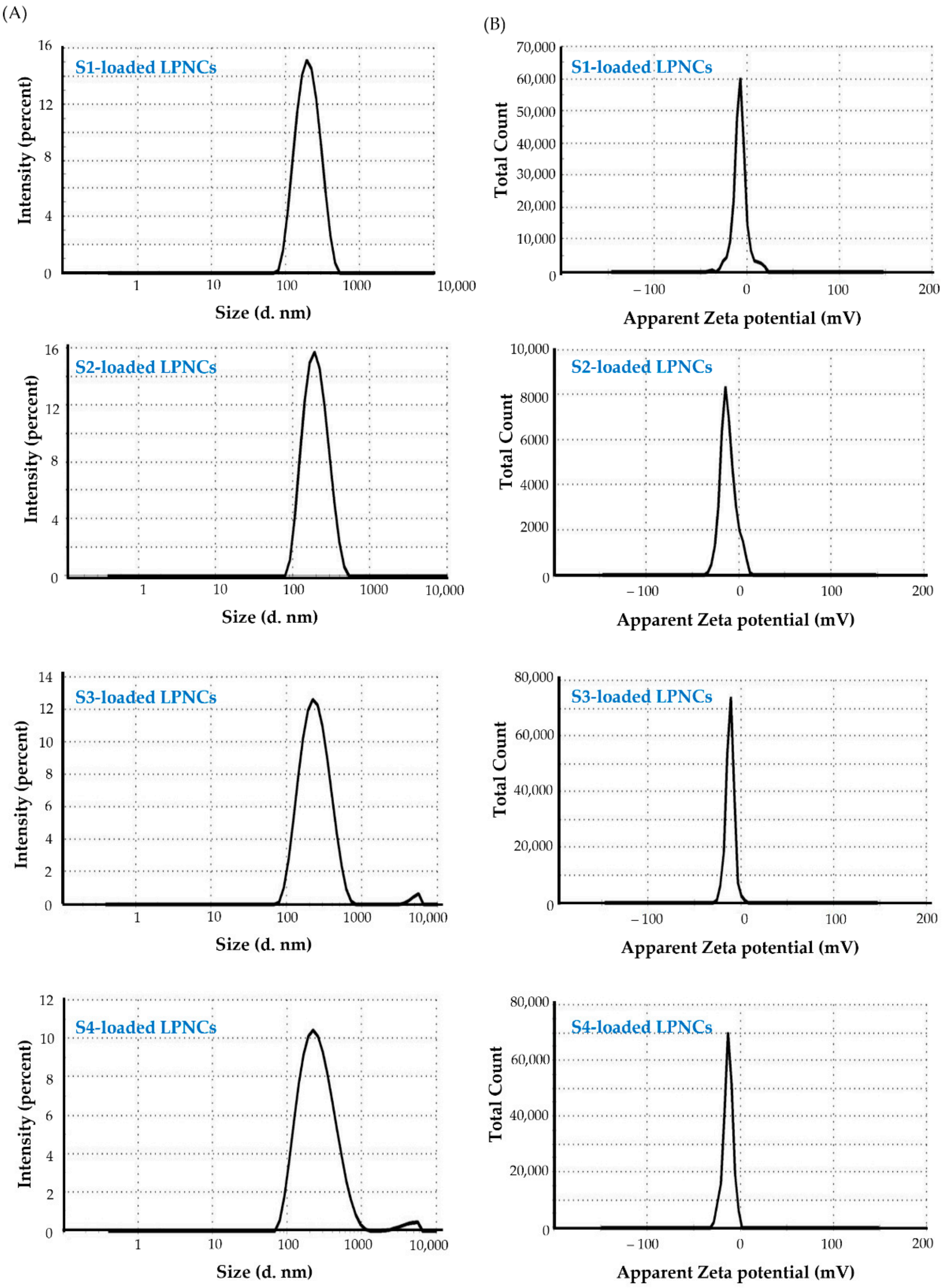

Figure S1. (A) Particle size and (B) zeta potential of various test compound-loaded LPNCs.

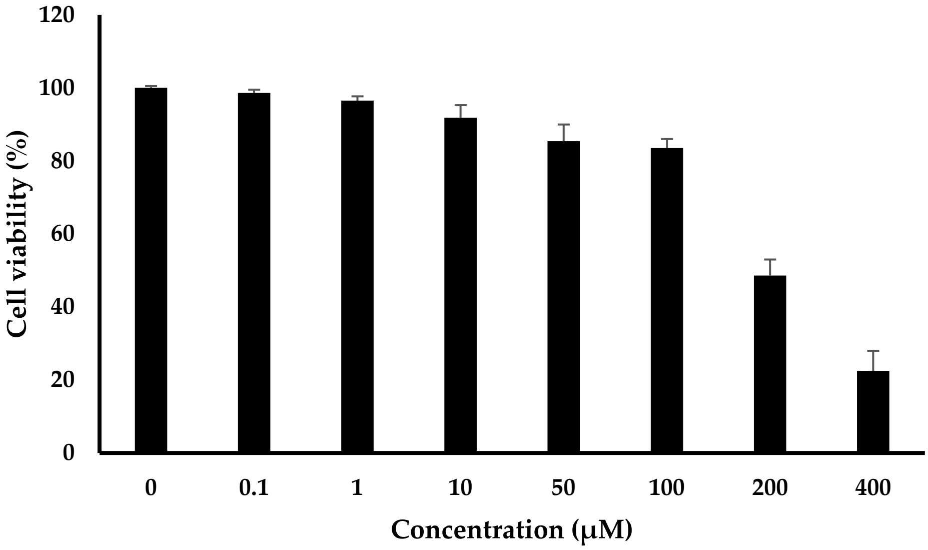

Figure S2. *In vitro* cytotoxicity of S4-loaded LPNCs against normal noncancerous MCF-10A breast cancer cells.
